# Maternal and perinatal outcomes of asthma exacerbation during pregnancy in a Chinese population: a retrospective cohort study

**DOI:** 10.1186/s12890-024-03112-8

**Published:** 2024-06-25

**Authors:** Tongxin Li, Fawu Dong, Nan Li, Chun Chang, Yan Wang

**Affiliations:** 1 Department of General Practice, Peking University Third Hospital, Beijing, 100191 China; 2 Department of Respiratory and Critical Care Medicine, Peking University Third Hospital, Beijing, 100191 China; 3 Clinical Epidemiology Research Center, Peking University Third Hospital, Beijing, 100191 China; 4 Center for Reproductive Medicine, Department of Obstetrics and Gynecology, PekingUniversity Third Hospital, Beijing, 100191 China; 5 National Clinical Research Center for Obstetrics and Gynecology, Beijing, 100191 China

**Keywords:** Asthma exacerbation, Pregnancy, Maternal and perinatal and outcomes

## Abstract

**Background:**

Asthma exacerbation (AE) is a significant clinical problem during pregnancy. This study aimed to identify maternal and perinatal outcomes associated with AE during pregnancy.

**Methods:**

We conducted a retrospective cohort study using the Peking University Third Hospital database from January 1, 2013 to December 31, 2020. We compared the clinical characteristics and maternal, perinatal and offspring outcomes of asthma with and without exacerbations among women who delivered during this period. The primary outcome was hypertensive disorders of pregnancy (HDP). Univariable and multivariable logistic regression analyses were used to analyze the clinical characteristics of AE during pregnancy and the association between AE and adverse maternal and perinatal outcomes.

**Results:**

The prevalence of asthma during pregnancy increased from 0.52% in 2013 to 0.98% in 2020. Of the 220 patients with asthma during pregnancy included in the study, 105 experienced AE during pregnancy: 62.9% (*n* = 66) had mild-to-moderate AE and 37.1% (*n* = 39) had severe AE. Pregnant women with allergic rhinitis have a higher risk of AE during pregnancy. Women who experienced AE were more at risk for hypertensive disorders of pregnancy than women who did not experience any exacerbation (12.4%vs3.5%, *p* < 0.05).

**Conclusions:**

The prevalence of asthma among pregnant women in China is on the rise. There is a notable correlation between pregnant women who suffer from allergic rhinitis and an elevated risk of AE during pregnancy. Studies have shown that AE during pregnancy are associated with an increased risk of hypertensive disorders of pregnancy.

**Supplementary Information:**

The online version contains supplementary material available at 10.1186/s12890-024-03112-8.

## Introduction

Asthma exacerbations (AE) are episodes marked by a progressive decline in lung function and increased asthma symptoms such as shortness of breath, coughing, wheezing, or chest tightness. When an AE occurs, airflow is restricted and airway becomes hyperresponsive, resulting in pathophysiological changes such as hypoxia. As our understanding of asthma management has grown, AE has been identified as a potentially major cause of adverse events. Asthma is a common chronic airway disease that affects 1–18% of the population in various countries [1]. In China, asthma is a major public health challenge, results from an extensive, comprehensive survey of a nationally representative sample indicated that asthma affects 4.2% of Chinese adults [2]. The prevalence of asthma in China is expected to rise rapidly [3].

Asthma is the most prevalent respiratory disorder that complicates pregnancy, and the prevalence of asthma in pregnancy was 0.3% in China [4, 5]. Asthma is linked to complications during pregnancy, poor perinatal outcomes, and allergic diseases in the offspring [6]. Approximately 33–45% of pregnant women with asthma experience AE that required medical intervention [7]. This increased the attention toward AE during pregnancy. Knowing whether adverse maternal and perinatal outcomes are related to AE is critical in managing asthma during pregnancy.

AE may increase the risk of preeclampsia, small for gestational age, low birth weight, and congenital abnormalities; however, the results have been inconsistent [8, 9]. Research on the perinatal outcomes in AE has been inconclusive, and more research is required to determine whether AE, particularly severe exacerbations, increases the risk of adverse outcomes. Preventing AE may be the key to reducing negative outcomes in future asthma management during pregnancy. Few relevant studies have been conducted in the Chinese population, particularly on the factors associated with the severity of AE and perinatal outcomes.

We hypothesize that asthma exacerbations during pregnancy are associated with an increased risk of HDP. This study established a retrospective cohort study to investigate the clinical characteristics and maternal and perinatal outcomes of AE to provide reference for the management of patients with asthma during pregnancy and insights for reducing the occurrence of AE and the associated adverse outcomes.

## Methods

### Study cohort and data collection

In this retrospective cohort study, pregnant women who delivered in the Department of Obstetrics of Peking University Third Hospital from January 2013 to December 2020 were included. To calculate the prevalence of pregnancy complicated by asthma, those diagnosed with asthma at discharge were included.

The exclusion criteria were as follows: (1) twin pregnancy; (2) for multiparous patients, any deliveries subsequent to their first were excluded; (3) autoimmune diseases requiring the use of glucocorticoidsoral glucocorticoids; and (4) incomplete clinical data.

The diagnosis of asthma was ascertained based on the discharge diagnoses recorded in the medical records. The ICD-10 clinical version code for Beijing is O99.502.

Data on patient clinical characteristics and asthma were obtained from medical records.

Clinical characteristics included maternal age, prepregnancy body mass index (BMI), smoking and drinking history, pregnancy weight gain, parity, thyroid disease, diabetes, hypertension, gastroesophageal reflux, allergic rhinitis, allergic conjunctivitis, drug and food allergy, atopic dermatitis, urticaria, eczema, and other complications.

The primary outcome was hypertensive disorders of pregnancy (HDP), including gestational hypertension, preeclampsia, and superimposed preeclampsia. Since we did not have any cases of eclampsia, it has not been included in our analysis. Perinatal outcomes included preterm birth (< 37 weeks’ gestation), small for gestational age (SGA)(< 10th percentile for gestational age). Offspring allergic diseases included eczema, atopic dermatitis, rhinitis, food allergy.

Obstetric characteristics included mode of delivery, gestational weeks (w), fetal sex and birth weight.

After obtaining verbal informed consent, telephone follow-up was used to gather information on the children’s long-term outcomes in order to analyze the influence of AE on their allergic diseases.

Telephone follow-up included the following questions by a physician: (1) Does your child have a doctor’s diagnosis of eczema or atopic dermatitis? (2) Does your child have a doctor’s diagnosis of asthma or rhinitis? (3) Does your child have a doctor’s diagnosis of food allergy?

The term “asthma worsening” was used to describe a brief emergence of asthma symptoms that did not necessitate a change in maintenance therapy. AE was defined as follows: (1) Mild-to-moderate exacerbation: Cases requiring an increase in maintenance medication for at least 2 days due to increased respiratory symptoms were deemed mild-to-moderate exacerbations [10]. (2) At least one of the following was present in a severe asthma exacerbation [11]: An emergency room visit or hospitalization due to asthma, the use of systemic corticosteroids (tablets, suspension, or injection), an increase from a stable maintenance dose for at least 3 days, or any of these conditions. Patients with asthma exacerbations during pregnancy who had occurred more than once were classified based on the most severe condition.

### Statistical analyses

The SPSS software version 26.0 was used for all statistical analyses, and a *P*-value of 0.05 was considered statistically significant. The Shapiro–Wilk test and Q–Q normality plots were used to check for data normality. Normally distributed continuous variables were displayed with means ± standard deviation and compared using t-tests. Non-normally distributed data were presented as the median (quartile) and were compared by a nonparametric test. The chi-squared test was used to compare categorical variables, expressed as frequencies (n) and proportions (%). The trend in the proportion of patients with asthma and AE was analyzed using the chi-square trend test. Univariable and multivariable logistic regression were used to analyze the clinical characteristics of AE. The models were adjusted for age, BMI, and odds ratios (ORs) were calculated with 95% confidence intervals (CIs). The Bonferroni method was used to correct for multiple testing. Univariate regression analyses for perinatal outcome were presented in Supplementary Tables S5, S6, and S7. The variables showing a significant association (*p*<0.2) and known confounders from previous studies were included in our multivariate regression models.

## Results

### Prevalence of asthma in pregnancy and asthma exacerbation in pregnancy

**Fig. 1:**
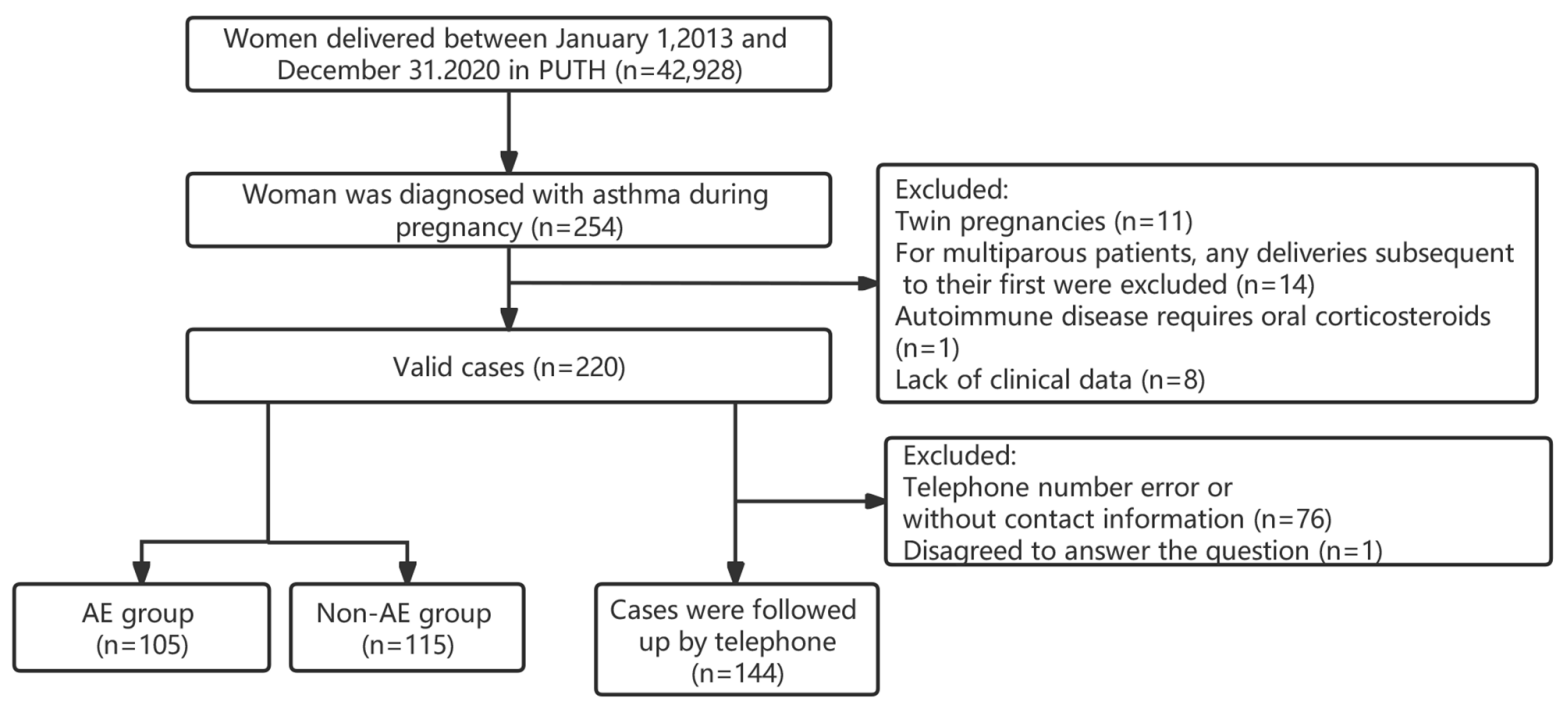
Flowchart of the participants included in the study. PUTH: Peking University Third Hospital; AE: asthma exacerbation

**Fig. 2:**
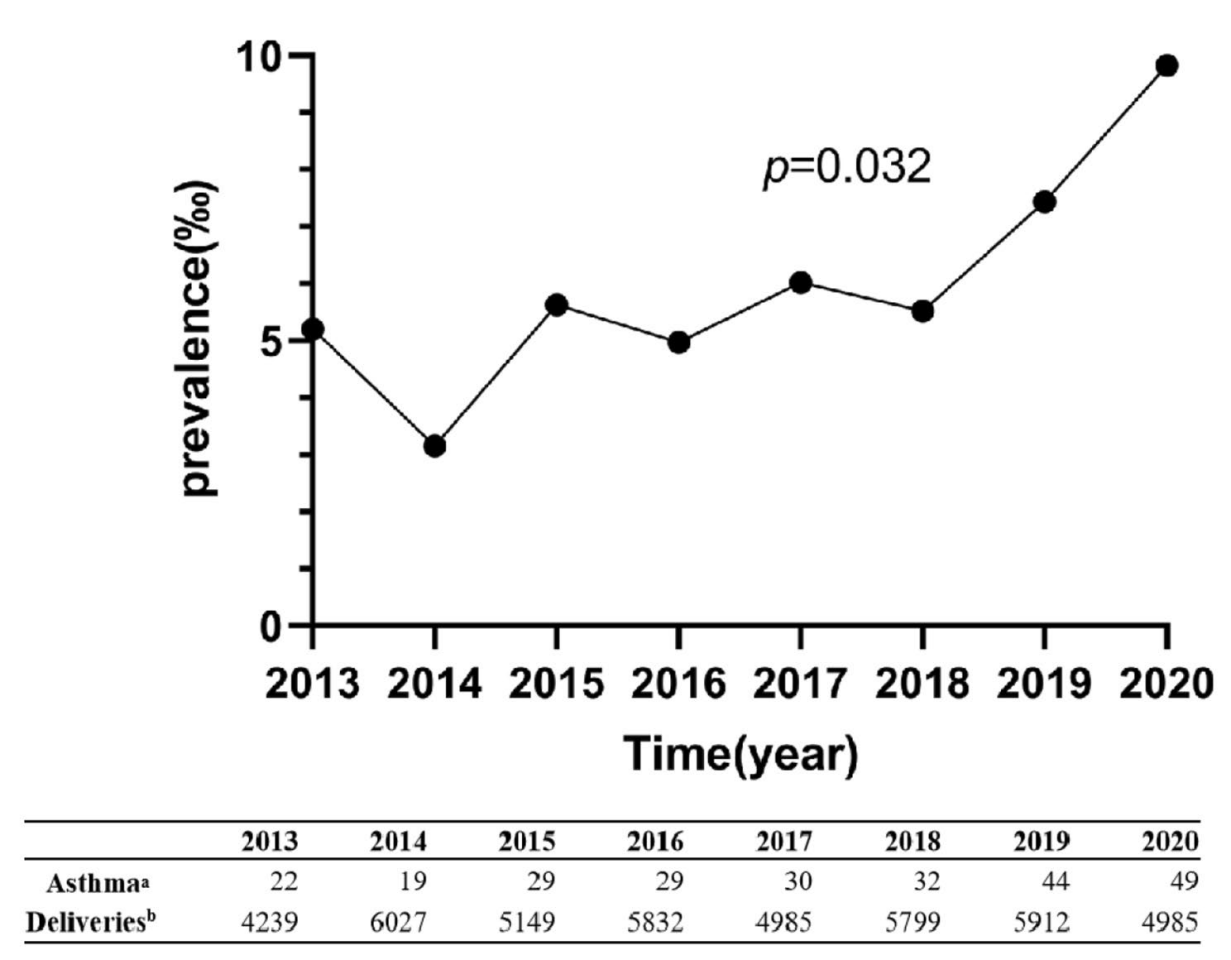
The prevalence of asthma in pregnancy. **a**: The number of asthmatic patients. **b**: The number of deliveries

From January 1, 2013, to December 31, 2020, 42,928 pregnant women delivered at the Third Hospital of Peking University. Of them, 254 patients were diagnosed with asthma during pregnancy (Fig. 1). The prevalence of asthma in pregnancy showed an upward trend with the change in years (*p* < 0.05): 0.52% in 2013, 0.32% in 2014, 0.56% in 2015, 0.50% in 2016, 0.60% in 2017, 0.55% in 2018, 0.74% in 2019, and 0.98% in 2020 (Fig. 2).

**Fig. 3:**
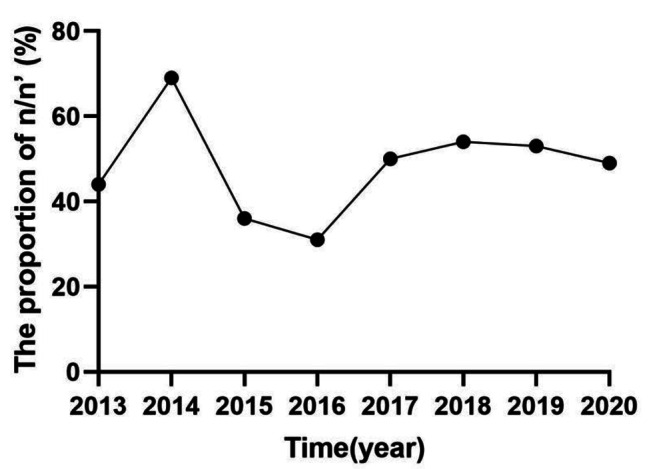
Percentage of patients with AE in pregnancy per year as a percentage of patients with asthma during pregnancy. n: The number of patients with AE during pregnancy. n’: The number of patients with asthma during pregnancy

In this study, 220 pregnant women, including 105 (47.7%) pregnant women with exacerbations and 115 (52.3%) pregnant women without exacerbations, were included. From 2013 to 2020, the proportion of AE in asthma during pregnancy fluctuated between 31% and 69% each year, and the proportion of AE in asthma during pregnancy was 48% (Fig. 3). Among the 105 pregnant women with AE, 39 cases (37.1%) experienced severe exacerbation, whereas 66 (62.9%) experienced mild-to-moderate exacerbation. In the severe AE group, 9 patients also experienced mild-to-moderate AE, while 30 were hospitalized in the emergency room or respiratory department due to AE.

AE occurred in a total of 131 times in 105 pregnant women with AE during pregnancy. Of them, 84 (80.0%) had one exacerbation, and 21 had at least two exacerbations (17 had two exacerbations, 3 had three exacerbations, and only 1 had four exacerbations).

**Fig. 4:**
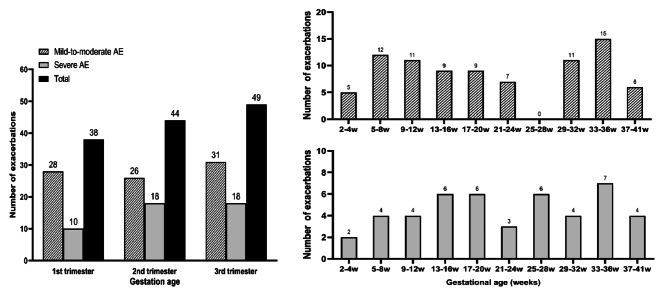
Distribution of AE during pregnancy in different gestational weeks

The rate of AE occurrence was 29.0%, 33.6%, and 37.4% during the first, second, and third trimesters, respectively (as shown in Fig. 4). The median value for each period is presented in Table S1.

### Clinical characteristics of asthma exacerbation during pregnancy

The proportion of allergic rhinitis was higher in the AE group than in the non-AE group (41.7% vs. 64.8%, *p* < 0.05). Neither group significantly differed in age, BMI, or weight gain during pregnancy. The incidences of thyroid disease, diabetes, hypertension, gastroesophageal reflux, and other conditions were similar between the two groups (Table 1).

**Table 1:** Clinical characteristics of pregnant women in the AE group and non-AE group

Clinical characteristics	Non-AE (*n* = 115)	AE (*n* = 105)	*P*-value
Maternal age (y)	33 (30,36)	32 (29,34.5)	0.126
BMI (kg/m^2^)	23.53 (20.83,25.28)	22.89 (20.83,25.87)	0.873
Weight gain (kg)	12.5 (10.0, 15.5)	13.5 (10,16)	0.200
Multiparous	39 (33.9)	34 (32.4)	0.809
Thyroid disease	17 (14.8)	15 (14.3)	0.917
Diabetes	1 (0.9)	2 (1.9)	0.607
Hypertension	3 (2.6)	5 (4.8)	0.394
Gastroesophageal reflux	5 (4.3)	5 (4.7)	0.883
Allergic rhinitis	48 (41.7)	68 (64.8)	0.001
Allergic conjunctivitis	9 (7.8)	12 (11.4)	0.364
Eczema	22 (19.1)	14 (13.3)	0.246
Atopic dermatitis	5 (4.3)	7 (6.7)	0.449
Urticaria	10 (8.7)	8 (7.6)	0.771
Drug allergy	26 (22.6)	14 (14.4)	0.165
Food allergy	5 (4.3)	9 (8.6)	0.200

### Maternal and perinatal outcome of asthma exacerbation during pregnancy

Women with AE during pregnancy were at a higher risk for hypertension disorders than those without AE (12.4% vs. 3.5%, *p* = 0.013)(Table 2). Furthermore, the AE group was subdivided into mild-to-moderate exacerbations and severe exacerbations. The incidence of hypertensive disorders of pregnancy (HDP) was 6.1% in the mild-to-moderate group and 17.9% in the severe group. When comparing the severe exacerbation group to the non-AE group, the incidence of HDP was significantly higher in the severe exacerbation group (17.9% vs. 3.5%, *p* < 0.001).

**Table 2:** Maternal and perinatal outcomes in women with asthma exacerbation during pregnancy

	Non-AE (*n* = 115)	AE (*n* = 105)	*P*-value
**Maternal outcome**			
HDP^#^ (primary)	4 (3.5)	13 (12.4)	0.013*
**Perinatal outcome**			
Preterm birth	8 (7.0)	12 (11.4)	0.249
SGA	6 (5.2)	5 (4.8)	0.877

^*^Significant after Bonferroni correction

#HDP, hypertensive disorders of pregnancy: gestational hypertension, preeclampsia, and superimposed preeclampsia

SGA, small for gestational age

Preterm birth and SGA were similar in both groups. The mean fetal weight was lower in women in the AE group than in the non-AE group (*p* < 0.05) (Table S4).

**Fig. 5:**
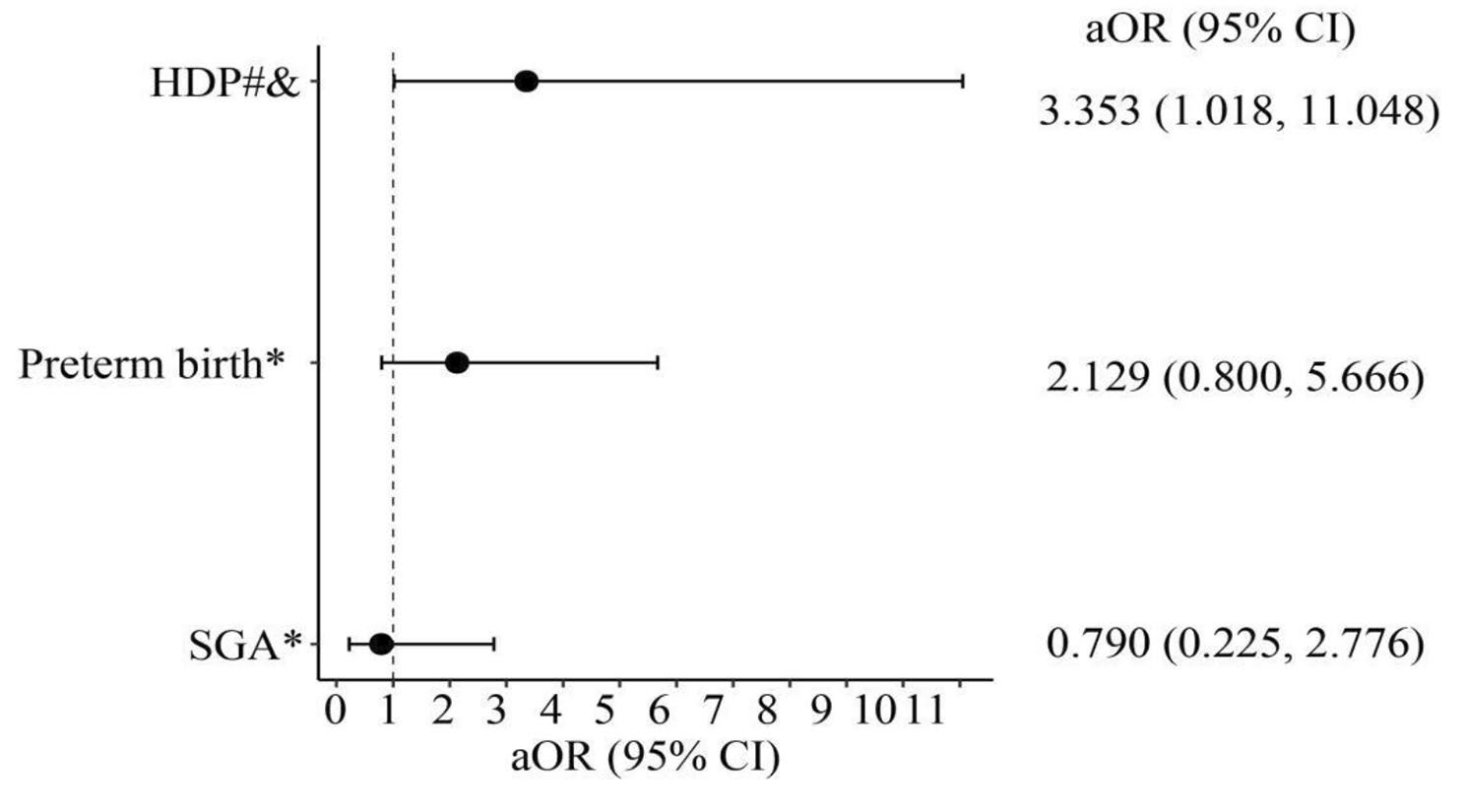
Risk of developing maternal and perinatal outcomes in women with asthma exacerbation during pregnancy. #HDP, hypertensive disorders of pregnancy: gestational hypertension, preeclampsia, and superimposed preeclampsia &Adjusted for age, BMI; *Adjusted for age, weight gain; aOR: Adjusted Odds ratio; CI: Confidence interval

Even after adjusting for confounding variables, AE remained an independent risk factor for hypertensive disorders of pregnancy (aOR 3.353, 95% CI 1.018–11.048). (Fig. 5).

### Effect of allergic diseases on children born to mothers experiencing asthma exacerbation during pregnancy

Among 220 pregnant asthmatic women, 144 cases completed the follow-up, and the follow-up rate was 65.5% (Fig. 1). Table S2 shows the prevalence of allergic diseases in the offspring of mothers with asthma during pregnancy in different age groups.

After adjusting for age, no significant difference existed between the non-AE, mild-to-moderate AE, and severe AE groups (Table S3).

## Discussion

The data of this study showed that asthma prevalence during pregnancy in the past 8 years was 0.59% and increased yearly, reaching 0.98% by 2020. The prevalence of exacerbations during pregnancy also showed an increasing trend, from 0.17% in 2013 to 0.42% in 2020. A large-scale study conducted across 20 municipal and higher institutions in 14 Chinese provinces reported in 2023 that the prevalence of asthma during pregnancy was 0.3% [5]. According to data from a South Korean population, 0.43% of pregnant women had asthma between 2009 and 2011 [12]. Although the prevalence rate in the center is lower than that in developed countries, the proportion of AE during pregnancy is similar to that reported in foreign studies. Exacerbation occurred in 48% of pregnant women with asthma in our study. Murphy et al. reported that 45% of women experienced AE requiring medical intervention during pregnancy [13]. China has lower adult asthma prevalence (4.2%) than certain wealthy nations, such as Australia (27.4%), the United Kingdom (22.6%), and the United States (7.6%) [2, 14]. The incidence of asthma during pregnancy coincides with the prevalence of asthma overall, although it is also less common in this study’s sample than in industrialized nations.

AE may occur at all stages of pregnancy. However, results of previous studies and domestic and foreign guidelines on the distribution of AE during pregnancy are inconsistent. In a study involving 47 patients with AE, the researchers found that AE most often occurred at 17–24 weeks, with an average onset gestational age of 20.8 weeks [15]. In another study [6], the average gestational age of a severe AE in 53 patients was 28.1 weeks, and the peak occurred at 14–24 weeks of gestation. The median gestational age of 34 patients with mild AE was 28.5 weeks, and the peak was 25–32 weeks of gestation. The 2022 GINA mentioned that AE during pregnancy mostly occur in the second trimester [1]. However, Chinese asthma guidelines note that the AE during pregnancy most often occurs in the 24th to 36th weeks of gestation [16]. Studying the gestational age distribution in AE is crucial to help clinicians for advising patients on preventing AE and provide clinical ideas for studying AE mechanisms during pregnancy.

Allergic diseases such as asthma, rhinitis, and eczema share several common pathways and proteins in the pathogenesis, such as the IL4 signaling and GATA3-related pathways [17]. In this study, 52.7% of pregnant women with asthma also had allergic rhinitis (64.8% and 41.7% in the AE and non-AE groups, respectively). In a FENO-based study [18] on asthma management during pregnancy in Australia, women with rhinitis had worse asthma control, worse quality of life, and more anxiety during asthma management during pregnancy than women without rhinitis and asthma, and improved asthma conditions under active asthma management was also related to improved rhinitis symptoms.

In the analysis of maternal and perinatal outcomes of AE during pregnancy, the AE group had significantly higher rates of hypertensive disorders during pregnancy than the non-AE group. After adjusting for age, BMI, AE remained a risk factor for hypertensive disorders of pregnancy, which is consistent with the research conclusion by Abdullah et al. [19]. A recent meta-analysis involving 20 studies showed that compared with asthmatic women without AE, asthmatic women with AE during pregnancy delivered SGA and low birth weight infants [20]. In this study, the neonatal birth weight for pregnant women with exacerbations was significantly lower than that of asthmatic women without exacerbations, which is consistent with our findings. The specific mechanisms underlying the association between asthma and hypertensive disorders of pregnancy remain unclear. One possible explanation is that the systemic inflammatory response accompanying asthma exacerbations leads to endothelial dysfunction and smooth muscle contraction in the blood vessels through the release of various inflammatory mediators, such as interleukin-6 and leukotrienes. This results in increased vascular resistance, thereby contributing to the development of hypertension [21]. Moreover, chronic hypoxia caused by asthma can lead to vascular remodeling, which may be a potential factor contributing to preeclampsia and gestational hypertension [22]. Anthor possible explanation is that medications used to treat asthma, especially glucocorticoids, might induce hypertension during pregnancy [23]. However, women who do not actively manage their asthma with treatments such as inhaled corticosteroids are at a higher risk of experiencing adverse maternal and perinatal outcomes [24].

In this study, after adjusting for offspring age, the incidence of allergic diseases between asthmatic mothers with and without exacerbations was similar. There are few reports on the long-term effects of AE during pregnancy, and the results are inconsistent. Abdullah et al. [19] reported that the offspring of mothers with AE during pregnancy had an increased risk of asthma in the first 5 years after birth. A cohort study of 26,265 singleton live births from 1990 to 2002 found that children of mothers with asthma had a slightly increased risk of atopic dermatitis than those of mothers without asthma, but the risk of allergic rhinitis was not increased. There was no correlation between the control and severity of asthma in mothers and the two atopic diseases in their offspring [25]. However, the case-control study by Martel et al. included 8226 offspring of asthmatic mothers and found a significantly increased risk of offspring asthma in mothers with severe and poorly controlled asthma during pregnancy [26]. A prospective cohort study observed that the incidence of both early-onset persistent and transitory asthma in children is increased by maternal uncontrolled and moderate-to-severe asthma during pregnancy [27]. Future large sample-size long-term follow-up cohorts in the offspring of asthmatic mothers are needed to determine whether the offspring mothers with AE during pregnancy are at increased risk for various diseases during childhood, infancy, and adulthood.

Twin pregnancies were excluded from this study because of the complex factors influencing the outcome of twin pregnancies. In addition, the same pregnant women with asthma may have given birth twice during the study period. Second multipara delivery was excluded in this study to eliminate the influence of potential confounding factors.

This study compared the clinical characteristics, risk factors, and clinical outcomes of women who experienced AE during pregnancy with those who had asthma without AE and graded the severity of asthma exacerbations, which has received little attention in previous studies and has not been reported in China.

There are still several limitations to this study: First, this study presents single-center data, which is not sufficient to reflect the prevalence of asthma in pregnant women and the characteristics of the population in Beijing or North China. However, a review of 8 consecutive years of data from the same center helps to show trends in disease occurrence. Second, due to the study’s retrospective nature, it was difficult to collect the characteristics of maternal lifestyle risk and socioeconomic factors, changes in management practices and healthcare policies over time, and there is insufficient information to assess the severity of asthma before pregnancy. In addition, the analysis of potential confounders is limited. Patients with asthma who have milder symptoms or limited access to medical care may not seek medical attention, possibly leading to the underreporting of AE. Third, there is a lack of information on the actual medication of pregnant women beyond the prescription, which makes it impossible to assess patients’ adherence. Despite these limitations, the current study, to some extent, reflects a real-world model of asthma-related health management in pregnant asthmatics. Therefore, this study positively impacts the prenatal counseling and pregnancy management of women with asthma during pregnancy. Looking forward, we aspire to design multicenter, large-scale, prospective studies in the future to address these limitations. With effective care and a multidisciplinary approach, AE can be prevented, and adverse maternal and infant outcomes can be minimized.

AE is an important event in poor asthma control, which should receive special attention in the management of pregnant women in China. Our population-based cohort study identified the detrimental effects of AE during pregnancy. Targeting women with asthma during pregnancy and ensuring appropriate asthma management and postpartum follow-up may help reduce the risk of pregnancy complications, adverse maternal and perinatal outcomes, and early childhood respiratory disorders.

### Electronic supplementary material

Below is the link to the electronic supplementary material.

Supplementary Material 1

## Data Availability

The datasets used and/or analyzed during the current study are available from the corresponding author on reasonable request.

## Abbreviations

AE: Asthma exacerbation
BMI: Body mass index
HDP: Hypertensive disorders complicating pregnancy
SGA: Small for gestational age
CI: Confidence interval
