# Supplementary material for: Maternal and perinatal outcomes of asthma exacerbation during pregnancy in a Chinese population: a retrospective cohort study
Source: BMC Pulm Med. 2024 Jun 25;24:296. doi: 10.1186/s12890-024-03112-8 (PMC11197224; doi:10.1186/s12890-024-03112-8)
Supplement: Supplementary file 1 — Supplementary Material 1 [file 12890_2024_3112_MOESM1_ESM.docx]

**Table S1:** Distribution of AE during pregnancy in different stages of pregnancy

|  | The first trimester | The second trimester | The third trimester | Total |
| --- | --- | --- | --- | --- |
| AE | 38 (29.0) | 44 (33.6) | 49 (37.4) | 131 (100.0) |
| Mild-to -moderate AE | 28 (32.9) | 25(30.6) | 32 (36.5) | 85 (64.9) |
| Gestational week(w) | 8.0 (5.0, 12.0) | 19.0 (15.0, 22.5) | 34.5 (31.0, 36.0) | 20.0 (11.5, 32.5) |
| Severe AE | 10 (21.7) | 19 (41.3) | 17 (37.0) | 46 (35.1) |
| Gestational week(w) | 7.0 (4.8, 10.5) | 20.0 (16.0, 24.0) | 33.0 (30.0, 37.0) | 22.5 (13.8, 32.3) |

Data presented as median (25th percentile; 75th percentile) and n(%). The first trimester (≤12+6 gestational weeks); The second trimester (13-27+6 gestational weeks) ; The third trimester (≥28 gestational weeks )

**Table S2:** The prevalence of allergic diseases in offspring（n=144）

|  | Total | Non-AE  n=74 | Mild-to -moderate AE  n=42 | Severe AE  n=28 |
| --- | --- | --- | --- | --- |
| Asthma | 12/144(8.3) | 7(9.5) | 4(9.5) | 1(3.6) |
| 0-5 (y) | 6(41.7) | 3(4.1) | 2(4.8) | 1(3.6) |
| 6-8 (y) | 6(41.7) | 4(5.4) | 2(4.8) | 0(0.0) |
| Rhinitis | 31/144(21.5) | 18/74(24.3) | 9/42(21.4) | 4/28(14.3) |
| 0-5 (y) | 17(11.8) | 10(13.5) | 4(9.5) | 3(10.7) |
| 6-8 (y) | 14(9.7) | 8(10.8) | 5(11.9) | 1(3.6) |
| AD/ Eczema | 53/144(36.8) | 27/74(36.5) | 16/42(38.1) | 10/28(35.7) |
| 0-5 (y) | 39(27.1) | 23(31.1) | 8(19.0) | 8(28.6) |
| 6-8 (y) | 14(9.7) | 4(5.4) | 8(19.0) | 2(7.1) |
| Food allergy | 30/144(20.8) | 18/74(24.3) | 7/42(16.7) | 5/28(17.9) |
| 0-5 (y) | 23(16.0) | 15(20.3) | 4(9.5) | 4(14.3) |
| 6-8 (y) | 7(4.9) | 3(4.1) | 3(7.1) | 1(3.6) |

**Table S3:** Relationship between AE and allergic diseases in offspring

| Allergic diseases | Non-AE  n=74 | AE  n=70 | *aOR (*95%*CI)* | Severe AE  n=28 | *aOR(*95%*CI)** |
| --- | --- | --- | --- | --- | --- |
| Asthma | 7/74(9.5) | 4/42(9.5) | 0.692(0.196-2.442) | 1/28(3.6) | 0.778(0.195-3.104) |
| Rhinitis | 18/74(24.3) | 13/70(18.6) | 0.666(0.276-1.610) | 3/28(10.7) | 0.787(0.292-2.120) |
| AD/Eczema | 27/74(36.5) | 26/70(37.1) | 1.001(0.522-2.026) | 10/28(35.7) | 1.072(0.490-2.346) |
| Food allergy | 18/74(24.3) | 12/70(17.1) | 0.645(0.284-1.463) | 5/28(17.9) | 0.605(0.228-1.606) |

*Adjusted for offspring age

AD: Atopic dermatitis

**Table S4:** Delivery information in women with asthma during pregnancy

|  | **Non-AE (n = 115)** | **AE (n=105)** | **P value** |
| --- | --- | --- | --- |
| Mode of delivery |  |  | 0.966 |
| Cesarean section | 50 (43.5) | 20 (51.3) |  |
| Vaginal delivery | 65 (56.5) | 19 (48.7) |  |
| Gestational weeks (w) | 39.43 (38.43,40.14) | 39.29 (38.29,39.71) | 0.220 |
| Infant (male) | 64 (55.7) | 23 (59.0) | 0.839 |
| Birth weight (g) | 3390 (3080, 3620) | 3260 (3070, 3550) | 0.010 |

**Table S5:** Univariate analysis of risk factors for HDP

|  | **Unadjusted OR** | **95%*CI*** | **P value** |
| --- | --- | --- | --- |
| Maternal age (y) | 0.942 | 0.825-1.075 | 0.376 |
| BMI (kg/m^2^) | 1.214 | 1.081-1.362 | 0.001 |
| Multiparous | 0.598 | 0.188-1.902 | 0.383 |
| Weight gain (kg) | 1.033 | 0.937-1.138 | 0.515 |
| Experienced AE during pregnancy | 3.921 | 1.236-12.436 | 0.020 |

HDP, hypertensive disorders of pregnancy: gestational hypertension, preeclampsia, and superimposed preeclampsia

**Table S6:** Univariate analysis of risk factors for preterm birth

|  | **Unadjusted OR** | **95%*CI*** | **P value** |
| --- | --- | --- | --- |
| Maternal age (y) | 1.050 | 0.939-1.174 | 0.394 |
| BMI (kg/m^2^) | 1.068 | 0.954-1.195 | 0.253 |
| Multiparous | 1.739 | 0.686-4.405 | 0.244 |
| Weight gain (kg) | 0.901 | 0.822-0.988 | 0.026 |
| Experienced AE during pregnancy | 1.726 | 0.676-4.404 | 0.254 |

**Table S7:** Univariate analysis of risk factors for SGA

|  | **Unadjusted OR** | **95%*CI*** | **P value** |
| --- | --- | --- | --- |
| Maternal age (y) | 0.840 | 0.696-1.013 | 0.069 |
| BMI (kg/m^2^) | 1.006 | 0.858-1.179 | 0.944 |
| Weight gain (kg) | 0.912 | 0.810-1.026 | 0.124 |
| Multiparous | 1.159 | 0.328-4.095 | 0.818 |
| Experienced AE during pregnancy | 0.908 | 0.269-3.069 | 0.877 |

SGA, small for gestational age
